# Supplementary material for: Multispecies and individual gas molecule detection using Stokes solitons in a graphene over-modal microresonator
Source: Nat Commun. 2021 Nov 18;12:6716. doi: 10.1038/s41467-021-26740-8 (PMC8602637; doi:10.1038/s41467-021-26740-8)
Supplement: Supplementary file 1 — Supplementary Information [file 41467_2021_26740_MOESM1_ESM.pdf]

Multispecies and individual gas molecule detection using Stokes solitons in a graphene over-modal microresonator

Teng Tan[1,2#], Zhongye Yuan[1#], Hao Zhang[1#], Guofeng Yan[2], Siyu Zhou[3], Ning An[1], Bo Peng[3], Giancarlo Soavi[4,5*], Yunjiang Rao[1,2*], Baicheng Yao[1*]

[1]*Key Laboratory of Optical Fiber Sensing and Communications (Education Ministry of China), University of Electronic Science and Technology of China, Chengdu 611731, China.*

[2]*Research Centre of Optical Fiber Sensing, Zhejiang Laboratory, Hangzhou 310000, China.*

[3]State Key Laboratory of Electronic Thin Film and Integrated Devices, University of Electronic Science and Technology of China, Chengdu 611731, China.

[4]Institute of Solid State Physics, Friedrich Schiller University Jena, Jena 07743, Germany.

[5]Abbe Center of Photonics, Friedrich Schiller University Jena, Jena 07745, Germany.

[#]These authors contributed equally.

*Corresponding authors: yaobaicheng@uestc.edu.cn; yjrao@uestc.edu.cn; giancarlo.soavi@uni-jena.de

1. Supplementary notes

S1. Co-generation and interaction of the Kerr and Stokes combs

We study the interaction of the external pump laser, the Kerr comb and the Raman process in our microsphere resonator using the nonlinear method of silica optical fibers[1]. For simplicity, here we only consider one mode. The total intracavity oscillating electrical field is $\mathbf{E}(t,\varphi) = \mathbf{E}_p + \mathbf{E}_k + \mathbf{E}_s$, and includes the slowly varying envelope of the external pump laser, the Kerr comb and the Stokes comb. Here t and φ refer to time and detuning respectively. The pump provides energy for both the four wave mixing and the stimulated Raman scattering processes[2]. Starting from the Lugiato-Lefever-equation (LLE), a pair of coupled equations in frequency domain describing the intracavity slowly-varying field can be extended by adding the Raman term[3]:

$$\omega E_k(\varphi) = \frac{D_{2k}}{2}\frac{\partial^2 E_k}{\partial \varphi^2} + (1-r)\left(\frac{n_2\omega_k D_{1k}}{2n\pi A_{kk}}|E_k|^2 + \frac{n_2\omega_k D_{1k}}{n\pi A_{ks}}|E_s|^2\right)E_k + \frac{rE_k}{D_{1k}}\int h_R\left(\frac{\varphi-\varphi'}{D_{1k}}\right)\left[\frac{n_2\omega_k D_{1k}}{2n\pi A_{kk}}|E_k(\varphi')|^2 + \frac{n_2\omega_k D_{1k}}{2n\pi A_{ks}}|E_s(\varphi')|^2\right]d\varphi' + \frac{rE_s}{D_{1k}}\int h_R\left(\frac{\varphi-\varphi'}{D_{1k}}\right)\frac{n_2\omega_k D_{1k}}{2n\pi A_{ks}}E_k(\varphi')E_s^*(\varphi')\exp\left(i\Omega\frac{\varphi-\varphi'}{D_{1k}}\right)d\varphi' - j\left(\frac{\kappa_k}{2} + j\Delta\omega_k\right)E_k + j(\kappa_e P_k)^{\frac{1}{2}} \quad \text{(S1)}$$

$$\omega E_s(\varphi) = j(D_{1k} - D_{1s})\frac{\partial E_s}{\partial \varphi} + \frac{D_{2s}}{2}\frac{\partial^2 E_s}{\partial \varphi^2} + (1-r)\left(\frac{n_2\omega_s D_{1s}}{2n\pi A_{ss}}|E_s|^2 + \frac{n_2\omega_s D_{1s}}{n\pi A_{ks}}|E_s|^2\right)E_s + \frac{rE_s}{D_{1k}}\int h_R\left(\frac{\varphi-\varphi'}{D_{1k}}\right)\left[\frac{n_2\omega_s D_{1s}}{2n\pi A_{ss}}|E_s(\varphi')|^2 + \frac{n_2\omega_s D_{1s}}{2n\pi A_{ks}}|E_k(\varphi')|^2\right]d\varphi' + \frac{rE_k}{D_{1k}}\int h_R\left(\frac{\varphi-\varphi'}{D_{1k}}\right)\frac{n_2\omega_s D_{1s}}{2n\pi A_{ks}}E_s(\varphi')E_k^*(\varphi')\exp\left(-i\Omega\frac{\varphi-\varphi'}{D_{1k}}\right)d\varphi' - j\left(\frac{\kappa_s}{2} + j\Delta\omega_s\right)E_s + jg_R P_s \quad \text{(S2)}$$

In contrast to Ref [3], in this model we consider strong Raman scattering, which can be excited directly by the pump laser. Here $\mathbf{E}_k$ and $\mathbf{E}_s$ are the normalized fields of the Kerr and Stokes comb, with discrete $\omega_{k,s}$, which could be written via Taylor expansion $\omega_u = \omega_0 + D_1 u + (1/2)D_2 u^2$......Here u is the mode number. $D_{1,ks}$ and $D_{2,ks}$ refer to the FSR and the second order dispersion at u = 0. $\Omega = \omega_{0,k} - \omega_{0,s}$ is the central frequency difference. Moreover, $\kappa_{k,s}$ is the cavity loss and $\Delta\omega_{k,s}$ is the detuning of the mode zero of the comb and the cold cavity resonance. κ_e is the cavity-bus coupling rate of the pump, $P_p = P_k + P_s$ is the total intracavity pump power. A_{mn} is the nonlinear mode area, defined as

$$A_{mn} = \frac{\iint |A_m|^2 dxdy \iint |A_n|^2 dxdy}{\iint |A_m|^2 |A_n|^2 dxdy} \tag{S3}$$

Here A_m and A_n are transverse distribution of the interacting modes. $h_R(\omega)$ is the stimulated Raman response, whose real part reflects the index modulation, while the imaginary part determines the Raman gain[1],

$$\begin{aligned} \delta_R &= rRe[h_R] \\ g_R &= 2\gamma r Im[h_R] \end{aligned} \tag{S4}$$

Here r = 0.18, and $\gamma = n_2\omega/(cA_{sk})$. In the case of lossless quasi-continuous approximation (*e.g.* in silica, the pulse durations, hundreds fs to ps, are much longer than the Raman response time, < 10 fs), we can simplify the coupled equations to:

$$\omega E_k(\varphi) = \frac{D_{2k}}{2}\frac{\partial^2 E_k}{\partial \varphi^2} + \left(\frac{n_2\omega_k D_{1k}}{2n\pi A_{kk}}|E_k|^2 + \frac{n_2\omega_k D_{1k}}{n\pi A_{ks}}|E_s|^2\right)E_k - 2D_{1k}E_k\frac{\partial\left\{\frac{n_2\omega_k D_{1k}}{2n\pi A_{kk}}E_k{}^2 + \frac{n_2\omega_k D_{1k}}{n\pi A_{ks}}E_s{}^2\right\}}{\partial\varphi} - jR\frac{\omega_k}{\omega_s}E_s{}^2E_k + j(\kappa_e P_k)^{\frac{1}{2}} \tag{S5}$$

$$\omega E_s(\varphi) = j(D_{1k} - D_{1s})\frac{\partial E_s}{\partial\varphi} + \frac{D_{2s}}{2}\frac{\partial^2 E_s}{\partial\varphi^2} + \left(\frac{n_2\omega_s D_{1s}}{2n\pi A_{ss}}|E_s|^2 + \frac{n_2\omega_s D_{1s}}{n\pi A_{ks}}|E_s|^2\right)E_s - 2D_{1k}E_k\frac{\partial\left\{\frac{n_2\omega_s D_{1s}}{2n\pi A_{ss}}E_s{}^2 + \frac{n_2\omega_s D_{1s}}{n\pi A_{ks}}E_k{}^2\right\}}{\partial\varphi} + jR|E_k|^2E_s - j\left(\frac{\kappa_s}{2} + j\Delta\omega_s\right)E_s + jg_R P_s \tag{S6}$$

Here $R = cD_{1k}g_R/4n\pi A_{ks}$. To analytically solve the equations, we use the approximation that the Kerr soliton and the Stokes soliton are formed independently in the same mode family (D_{1k}-D_{1s}) = 0, the pump laser power is high and the nonlinear conversion efficiency is low (< 10^{-2}). First we use the sech^2 DKS model to calculate the Kerr comb evolution[4]. From this we obtain the $E_s(t,\varphi)$, assuming the mode Q factor 3×10^8, D_1 = 100 GHz, D_2 = -10 fs^2/mm. The calculated result is shown in **Fig. S1a and 1b**. During the evolution, when the Kerr soliton has just formed, the phase-locked four-wave-mixing would suppress the Raman scattering[5], because g_R is proportional to $1/\Delta k$ (Δk is the phase mismatching, when it approaches 0, g_R becomes very small). Hence the threshold of Stokes comb increases dramatically[6].

Fig. S1c illustrates the measured intracavity intensity evolution of the Kerr and Stokes combs, which are traced synchronously by using a C+L/U band wavelength division multiplexer. When red scanning the pump laser (at fixed power 200 mW) from 1549 nm to 1550 nm with a scanning speed of 500 MHz/ms, a Kerr comb begins to form and creates Raman amplification. To observe both Kerr and Stokes solitons, the following four conditions must be satisfied: 1) the Stokes soliton lines must lie within the Raman gain spectrum generated by the Kerr soliton; 2) the FSR of the Stokes solitons must be close to that of the Kerr

soliton; 3) the mode family of Kerr soliton and Stokes solitons overlap efficiently in both space and time; 4) the pump power reaches the Raman threshold. In this way, the Stokes solitons rely on the existence of the Kerr soliton due to the spatial-temporal overlap for Kerr effect trapping and Raman amplification. Here one can also observe that the Kerr soliton step appears earlier than the Stokes soliton step.

S2. Principles of the multiple heterodyne based on Stokes combs

In a multimode microresonator, multiple Stokes lines at different mode families can be generated since the SRS process doesn't require strict phase matching. By using the finite element method (FEM), we simulate the mode field distribution of several typical modes in our microsphere, as shown in **Fig. S2a**. In this simulation, we use the following parameters: microsphere diameter 600 μm, silica material index 1.446, central wavelength 1550 nm. It is clear that a higher order mode has a larger mode volume. When we deposit a layer of graphene a bit far away from the equator, the graphene can only interact with light in higher order modes. Based on the FEM simulation, we also include the group index n_G of these modes, as **Fig. S2b** shows. $n_G(\omega)$ determines the FSR (or the D_1)

$$FSR = \frac{c}{n_G L} \tag{S7}$$

Here L is the cavity perimeter, and $dFSR/d\omega$ determines the chromatic dispersion (D_2). Specifically, n_G from TE_{01} to TE_{05} mode, TM_{01} to TM_{05} mode decreases with the increasing mode order. In practice, there are much more transverse mode families, which can enable rich mode-to-mode interactions and beating. According to Ref [3], a condition for Stokes soliton and Kerr soliton co-locking is that the Stokes lines feature an FSR that is close in value to that of the Kerr soliton. Hence the locked Stokes soliton mode should be lower in order than the Kerr soliton mode. For instance, **Fig. S2c** plots the n_G dispersion curves of TE_{01} to TE_{05} mode, and TM_{01} to TM_{05} mode. For example, when the pump mode of a Kerr soliton is TM_{05}, the possible Stokes soliton could belong to TM_{04} (at 1597 nm), TE_{04} (at 1629 nm), TM_{03} (at 1642 nm), TE_{03} (at 1677 nm), and TM_{02} (at 1692 nm). Besides, when the pump mode of a Kerr soliton is TM_{01}, only the TE_{01} mode at 1586 nm shares the same n_G (or D_1). This indicates that multiple Stokes solitons with different *FSR* could co-exist. Specifically, in the case of pumping the TM_{05} mode at 1550 nm (locking FSR 108 GHz), we illustrate a calculated multi-Stokes soliton spectrum in **Fig. S2d**. The Stokes lines generated in different mode families clearly overlap and thus one can measure their beating (**Fig. S2e**).

S3. Device and gas sensing preparation

Fig. S3a sketches the fabrication process of our graphene covered microsphere device. We prepare the whisper-gallery-mode microresonator samples from standard silica fiber by using the arc-discharge fusing method. First, we put a silica fiber in a programmable fiber fusion splicer (FITEL S184). By controlling the arc discharge power, discharge duration and discharge position, we fabricate microspheres with the diameter of the ≈ 620 μm, whose scale error is less than 2 μm. For soliton comb formation, these samples can produce repetition rate ≈ 100 GHz. **Fig. S3b** shows the optical microscope image and scanning electron microscopic image of a fabricated microsphere sample, verifying that its surface is smooth, enabling ultrahigh *Q* factors. On the other hand, we prepare the high-quality crystalline graphene via PDMS based mechanical exfoliation[7]. Then, by using the dry-transfer technique, we deposit the graphene layer on the surface of the microsphere. In this process, we carefully control the graphene

position, making sure that intracavity transmitted light can interact with it although far from the equator. This also avoids risk of burning.

Fig. S3c plots the Raman spectrum of our graphene deposited on a silica microsphere. This spectrum is measured using a Renishaw InVia spectrometer, with excitation wavelength at 514 nm. The on-sample power is less than 0.5 mW, to avoid heating. In the Raman spectrum, there is almost no D peak and intensity ratio of the G/2D peak is ≈ 60%. The position of the G peak and the 2D peak are 1584 nm (linewidth 18 cm^{-1}) and 2688 nm (linewidth 25 cm^{-1}), respectively. These data indicate that the exfoliated graphene is almost defect-free. The D+D'' peak at 2450 cm^{-1} mainly reflects the fact that graphene is lying on a silica substrate rather than suspended[8]. According to the Raman spectroscopy, we confirm that the initial Fermi level of the graphene is ≈ 0.2 eV (p-doping).

The gas sample preparation process is shown in **Fig. S3d**. We prepare pure NH_3 gas (molecular weight 17 g/mol, density 0.76 g/L) at room temperature (≈ 300 K) with standard atmospheric pressure (101 kPa). The typical gas mole density is 0.045 mol/L under this condition. Then we dilute the NH_3 in dry air, by using a 1 mL chamber and a 1 rheodyne with resolution 1 μL (NH_3 is 4.5×10^{-8} mol per μL). After this process, we extract 1 μL of the diluted gas sample, in which the NH_3 concentration is 4.5×10^{-11} mol. Then we inject the diluted NH_3/Air hybrid into the sensing chamber (with the graphene microresonator inside, volume 8 L, gas number 0.357 mol). The minimum NH_3 concentration in the sensing chamber can reach 1.26×10^{-10}, or 0.126 ppb.

S4. Linear transmission of the microresonators

We characterize our microresonator samples by using a tunable laser (Santec TSL-710). The launched-in laser power is 0.2 mW, much lower than any nonlinear threshold. **Fig. S4a** sketches the setup for measuring the linear transmission. Here the laser scanning speed is 100 GHz/s, and the maximum sampling rate of the oscilloscope is 10^9 per second. Hence the maximum point-to-point spectral resolution is 100 Hz, enough for the resonance characterization. In **Fig. S4b**, we plot the measured cavity transmission over one FSR (1550 ~ 1551 nm). By carefully tuning the distance of the tapered fiber and the microresonators, we can enable the microresonators to work at the critical coupling point (intracavity loss = coupling loss)[9] of the fundamental mode (TM_{01}). In this case, most of other high order modes are in the over coupling region (intracavity loss < coupling loss). Due to its large mode volume, a > 600 μm microsphere supports many transverse mode families. As a result, we can see many resonant dips in one fundamental FSR. Moreover, after graphene deposition, due to the breaking of geometric symmetry, more high order modes are enhanced. The top panel in **Fig. S4c** counts the numbers of longitudinal mode families in one FSR, before and after graphene deposition. In a typical silica microsphere sample, we find 496 resonances, while we find 523 resonances after graphene deposition. This result also depends on the polarization. In the bottom panel, we show the coupling curves of two modes (TM_{01} and a high order mode H).

$$T = \frac{(\rho-|t|)^2}{(1-\rho|t|)^2} \tag{S8}$$

Here $\rho = 1-\alpha$ is the intracavity transmission, $t = 1-\kappa$ is the bus transmission. When $\rho = t$, a mode is at the critical coupling point. Calculated Q factors of these two modes are 3×10^8 (TM_{01}) and 6.1×10^7 (H). This H mode could be affected by the graphene (graphene introduces additional loss), but the light field

of the fundamental mode doesn't interact with the graphene. This is also the reason why the resonant dips of high order modes demonstrate larger depth in graphene microsphere. **Fig. S4d** plots the single resonance of two specific resonances (a fundamental mode and a higher order mode). The blue curves show the case before graphene deposition, and the red curves show the case after graphene deposition. The linewidth of the fundamental mode remains ≈ 0.65 MHz, suggesting an intrinsic Q factor 3×10^8. Due to the graphene deposition, the linewidth of the H mode increases from ≈ 1 MHz to ≈ 1.4 MHz, suggesting that its intrinsic Q factor decreases from 2×10^8 to 1.3×10^8.

S5. Co-formation of multiple soliton combs and the selected comb beats note.

Fig. S5a shows the experimental setup for comb generation. For the excitation we used a tunable ECDL (external cavity diode laser, Santec TSL-710, typical linewidth 200 kHz, tunable range 1480 ~ 1640 nm). After amplification by a high power EDFA (erbium doped fiber amplifier), the pump power can reach > 3 W (in practice, 200 mW after the EDFA is enough for Kerr and Raman soliton generation). Then we use a FPC (fiber polarization controller) to tune the light polarization and to optimize the fiber-to-cavity coupling. Such coupling is achieved by using a tapered fiber with diameter ≈ 1 μm. The microsphere device is carefully fixed in a chamber, and we use a TEC (thermal electrical cooler, Thorlabs) to accurately control the temperature. The generated combs could be separated by using a C/L band fiber based WDM (wavelength division multiplexer, 1625 nm) and measured in OSA (optical spectrum analyzer, Yokogawa 6370D), power meter (PM, data tracing) and ESA (electrical spectrum analyzer, R&S 0 ~ 43 GHz).

Fig. S5b plots three spectral frames during the comb evolution. All the nonlinear effects (including Kerr and Raman) are driven by the tunable ECDL pump. In the detuning process, we control the pump laser blue-to-red tuning manually. By increasing the red-detuning, we can obtain the 'unlocked Kerr + Stokes' state, then the 'Kerr soliton + suppressed Stokes' state, and finally the 'Kerr soliton + Stokes solitons' state. This result agrees well with the theoretical analysis. We note that the excited Stokes lines could belong to different mode families, as the stimulated Raman scattering does not require strict phase matching like FWM. When the Stokes solitons are locked on the Kerr soliton, they share a quasi-equal line-to-line space (≈ 100 GHz) but have different central frequency of the envelope. This enables detectable inter-comb beating down conversion. For sensing, we select overlapping Stokes solitons with proper frequency offsets, such as a 7.514 MHz signal coming from the heterodyne of $TE_{0,A+1}$ and $TM_{0,A}$ (RI difference 0.001). To satisfy the Kerr-Stokes co-locking, the pumping mode around 1550 nm should be in higher order (> A). In **Fig. S5c** and **S5d,** we measure the long-term stability of the 7.514 MHz beating signal. For a continuous measurement of 2 hours at room temperature the frequency shift is < 2.5 Hz and the intensity variation is < ± 0.1 dB. Such a high stability with uncertainty at the Hz level offers a unique platform for gas sensing applications.

S6. Optoelectronic property of graphene and quantitative discussion of molecular sensing

The carrier density of graphene determines its conductivity. In particular, the intra-band conductivity and the inter-band conductivity can be written as [10]

$$\sigma_{g,intra} = \frac{ie^2 E_F}{\pi\hbar(2\pi f + \frac{i}{\tau})} \quad \text{(S9)}$$

$$\sigma_{g,inter} = \frac{ie^2 E_F}{4\pi\hbar} ln\left[\frac{2|E_F| - \hbar(2\pi f + \frac{i}{\tau})}{2|E_F| + \hbar(2\pi f + \frac{i}{\tau})}\right] \tag{S10}$$

Here E_F is the quasi-Fermi level, directly determined by the external bias. f is the optical frequency, $\tau \approx 10^{-13}$ s is the carrier relaxation lifetime, $\hbar = 1.05\times10^{-34}$ J·s is the reduced Planck constant, and $e = -1.6\times10^{-19}$ C is the unit charge. External electric tuning (such as gas adsorption) mainly influences the $\sigma_{g,inter}$, which is also directly related to the saturable absorption. Considering graphene as an atomically thick planar waveguide with such complex sheet conductivity, we can write its effective optical permittivity as[11]

$$\epsilon_g = \frac{-Im(\sigma_{g,i}) + iRe(\sigma_g)}{2\pi f\Delta} \tag{S11}$$

Here Re(σ_g) and Im(σ_g) are the real and imaginary parts of the σ_g respectively. By considering a thickness $\Delta = 0.33$ nm, the refractive index of the graphene layer can be derived from

$$\{Re(n_g) + iIm(n_g)\}^2 = Re(\epsilon_g) + iIm(\epsilon_g) \tag{S12}$$

For media modes with real permittivity, we can calculate

$$\begin{cases} Re(n_g) = \dfrac{2Re(\epsilon_g)\left(\frac{Re(\epsilon_g)}{2} + \frac{\sqrt{Re(\epsilon_g)^2 - Im(\epsilon_g)^2}}{2}\right)^{1/2} - 2\left(\frac{Re(\epsilon_g)}{2} + \frac{\sqrt{Re(\epsilon_g)^2 - Im(\epsilon_g)^2}}{2}\right)^{3/2}}{\epsilon_{g,i}} \\ Im(n_g) = \left(\dfrac{\sqrt{Re(\epsilon_g)^2 + Im(\epsilon_g)^2}}{2} - \dfrac{Re(\epsilon_g)}{2}\right)^{1/2} \end{cases} \tag{S13}$$

Fig. S6 maps the calculated spectra of the complex ϵ_g and n_g, for different Fermi levels. This shows that both the real and the imaginary parts of the graphene's refractive index can oscillate dramatically during the gate tuning. For a specific optical wavelength at 1550 nm (our Kerr comb region), the maximum tuning range of the Re(n_g) is from 0.20 to 3.23, while the Im(n_g) is from 0.25 to 1.42.

In this case, we can quantitatively estimate the soliton-soliton offsets induced by an individual molecule adsorption event on the graphene surface. The graphene's Fermi level is given by $|E_F| = \hbar|v_F|(\pi N)^{1/2}$ *with* $\hbar = 6.582\times10^{-16}$ eV s. In our case, we have a carrier density N = 2.94×10^{16} m^{-2} (E_F = 0.2 eV on silica) and an exposed area of 2×10^{-9} m^2. Let's now use single molecule NH_3 detection as an example. Each NH_3 molecule adsorption event will result in the transfer of two electrons from NH_3 to graphene, thus inducing a change in its carrier density and Fermi level $\Delta|E_F| = \hbar|v_F|(\pi)^{1/2}\{(N_1)^{1/2}-(N_2)^{1/2}\}$ $\approx 4\times10^{-9}$ eV per molecule. If we now consider a wavelength ≈ 1650 nm, we obtain that the real part of graphene's refractive index n_g increases approximately by 0.8/eV [12], and thus the n_g increment is $\approx 3\times10^{-9}$. Considering a typical beat note f_A = 7.51 MHz from the overlap between graphene and the mode pair TM_{04} and TE_{05}, the adsorption of individual NH_3 molecules will induce an effective group index variation of $\approx 3\times10^{-13}$. Here the n_g dependent effective group index can be calculated via a commercial software (*COMSOL Multiphysics*) using the finite element method (See supplementary note S2). Hence, the spectral variation of its soliton-soliton offset is ≈ 0.02 Hz per molecule (Eq. S7). Since the lock-in amplitude-frequency relationship is 10 mV/Hz, the adsorption of an individual NH_3 molecule will induce a step of ≈ 0.2 mV. This estimate is in excellent agreement with our experimental result.

S7. Optoelectronic implementation for gas tracing

Fig. S7a shows the experimental setup for gas sensing measurement. The device is put in a gas chamber equipped with optical fiber coupling and a temperature controller. The generated Stokes solitons are checked with an OSA, ensuring that they are in locked state. Then the optical signal is detected by using an amplified photodetector (Thorlabs APD 103C), that we use to monitor their beat notes. The beat notes are detected in an ESA (R&S, 0 ~ 43 GHz). For further amplification of the electrical signals and to match the bandwidth of our lock-in amplifier (Stanford Research, SR 810, 125 kHz), we use a RF signal generator (Tektronix TSG4100A, 0 ~ 200 MHz) to create a reference signal for further down-conversion in a frequency mixer. **Fig. S7b** plots the two RF signals. The frequency difference between the reference and our selected comb beat note is tunable (we commonly use the number 50 kHz). Mixing of the two lines (with frequency f_A = 7.5 MHz, and f_B = 7.55 MHz) generates more frequency components: $f_B - f_A$, $f_B + f_A$, and their harmonics such as $2f_A - f_B$, $2f_B - f_A$, $3f_A - 2f_B$, $3f_B - 2f_A$, etc. Specifically, we only amplify and trace the down conversion signal with f_d = 50 kHz (**Fig. S7c**). Thanks to the stable nature of the soliton and the amplification during the electrical mixing, SNR of this 50 kHz signal is > 70 dB, with 3dB linewidth < 2 Hz, as shown in the zoomed-in panel (band: 49.9 kHz to 50.1 kHz). In the lock-in implementation, we tune the lock-in filter band to 3 Hz (integration time 300 ms) at 50 kHz, to obtain enough amplification rate and enhance the sensitivity. When the electrical signal is stable, the output of the lock-in amplifier is a CW wave, which is monitored in an oscilloscope. Once gas molecules are adsorbed on the graphene-microsphere device, the 50 kHz signal will shift and we will detect a large change in intensity. **Fig. S7d** compares the sensitivity of the microsphere Stokes comb device, before and after graphene deposition. The Stokes solitons generated in a pure silica microresonator show no sensitivity to gas molecules, as the NH_3 molecules cannot be adsorbed on silica.

2. Supplementary Figures

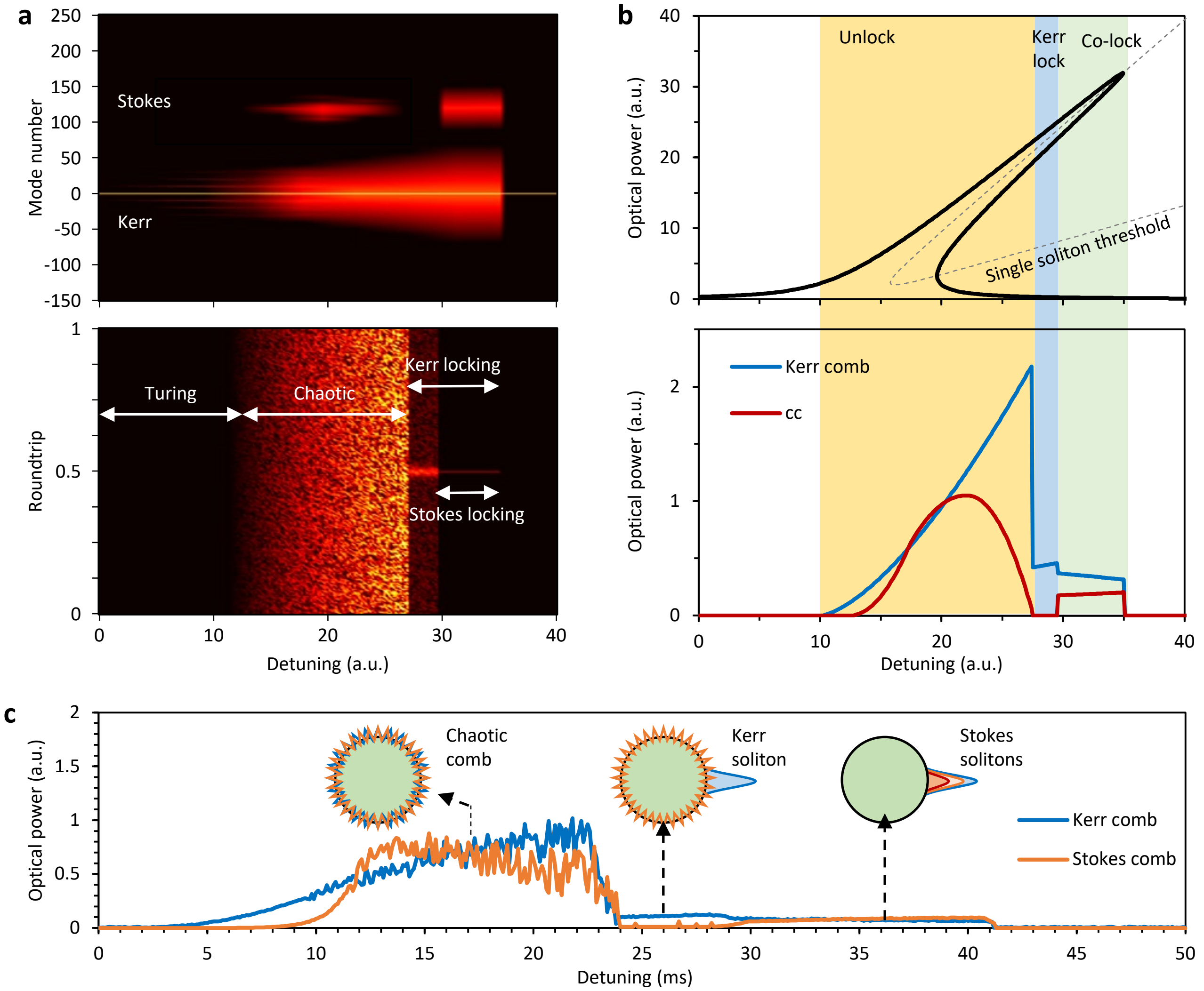

Fig. S1. Calculated results: Kerr and Stokes soliton evolution in a microresonator. a, Simulation of the intracavity field in frequency (upper panel) and time (bottom panel) domain. Here the detuning is normalized to the resonance. The Kerr single soliton appears after Turing state and chaotic state. The Stokes comb appears first, driven by the pumping laser. Then it is suppressed in the Kerr soliton region. Once the Kerr soliton power reaches the injected-locking threshold, the Stokes soliton is formed. **b,** Calculated curves. Upper panel: The Kerr triangle (black solid) and the first-order FWM bi-stable threshold (grey dashed). Bottom panel: Correlation of the detuning and the optical power, for the Kerr comb (blue) and Stoke comb (red). In the above simulations, we only calculated one Stokes mode (TM_{01}). **c,** Measured evolution of the Kerr (blue curve) and the Stokes (red curve) combs.

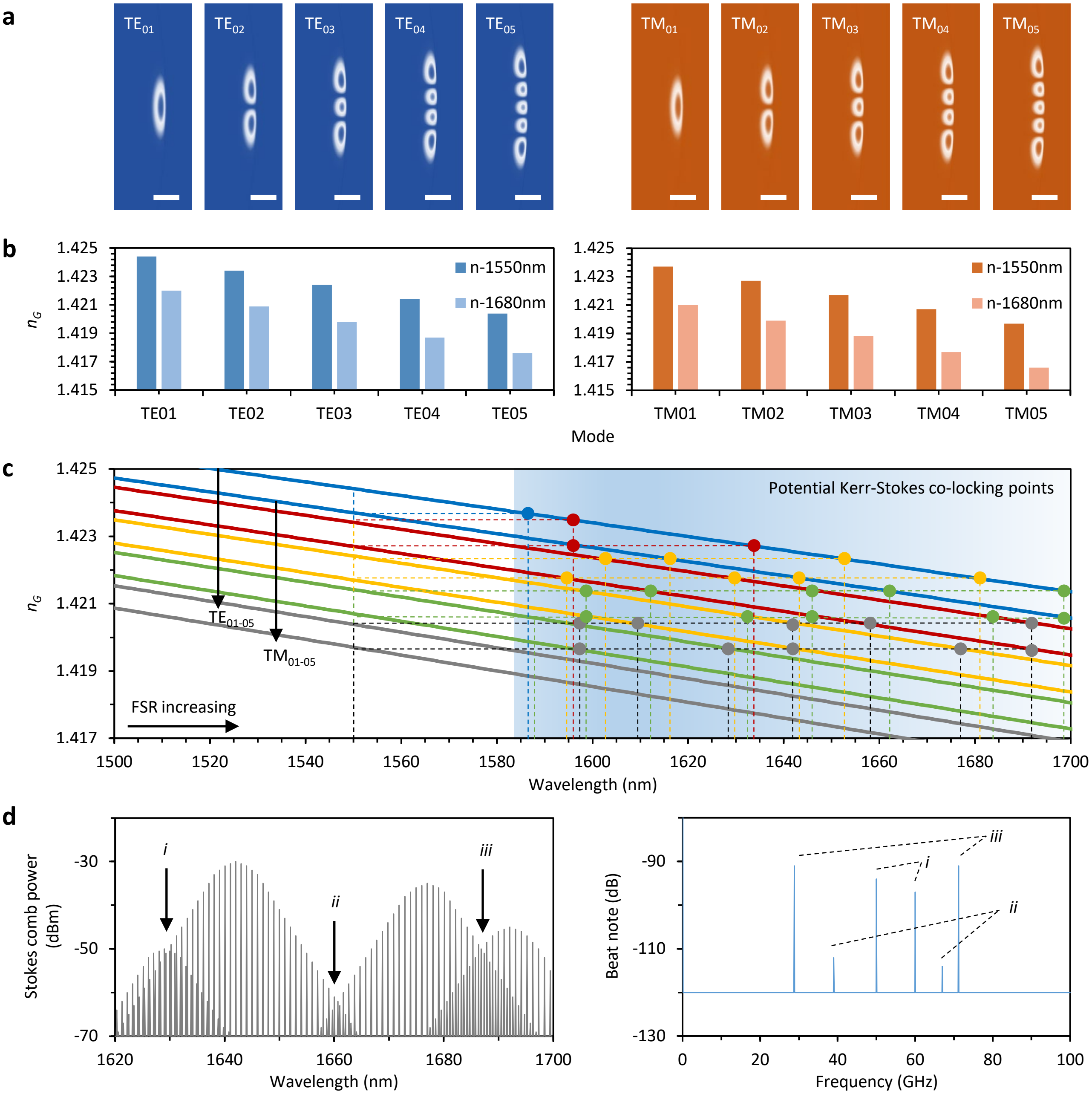

Fig. S2. The mode families of a multimode microresonator. a, Simulated electric field distributions. Left panels: Transverse electrical modes, order 01 to 05. Right panels: Transverse magnetic modes, order 01 to 05. **b,** Calculated n_G of the ten modes. **c,** n_G offset varies with wavelength. The n_G of TE_{01}/TM_{01}, TE_{02}/TM_{02} TE_{03}/TM_{03}, TE_{04}/TM_{04}, TE_{05}/TM_{05} are shown in blue, red, yellow, green and grey respectively. **d,** Spectrum of multiple Stokes solitons, in different modes (from left to right: TE_{04}, TM_{03}, TE_{03}, TM_{02}). **e,** Overlapping of the multiple combs enables beat notes with frequencies smaller than the resonator FSR.

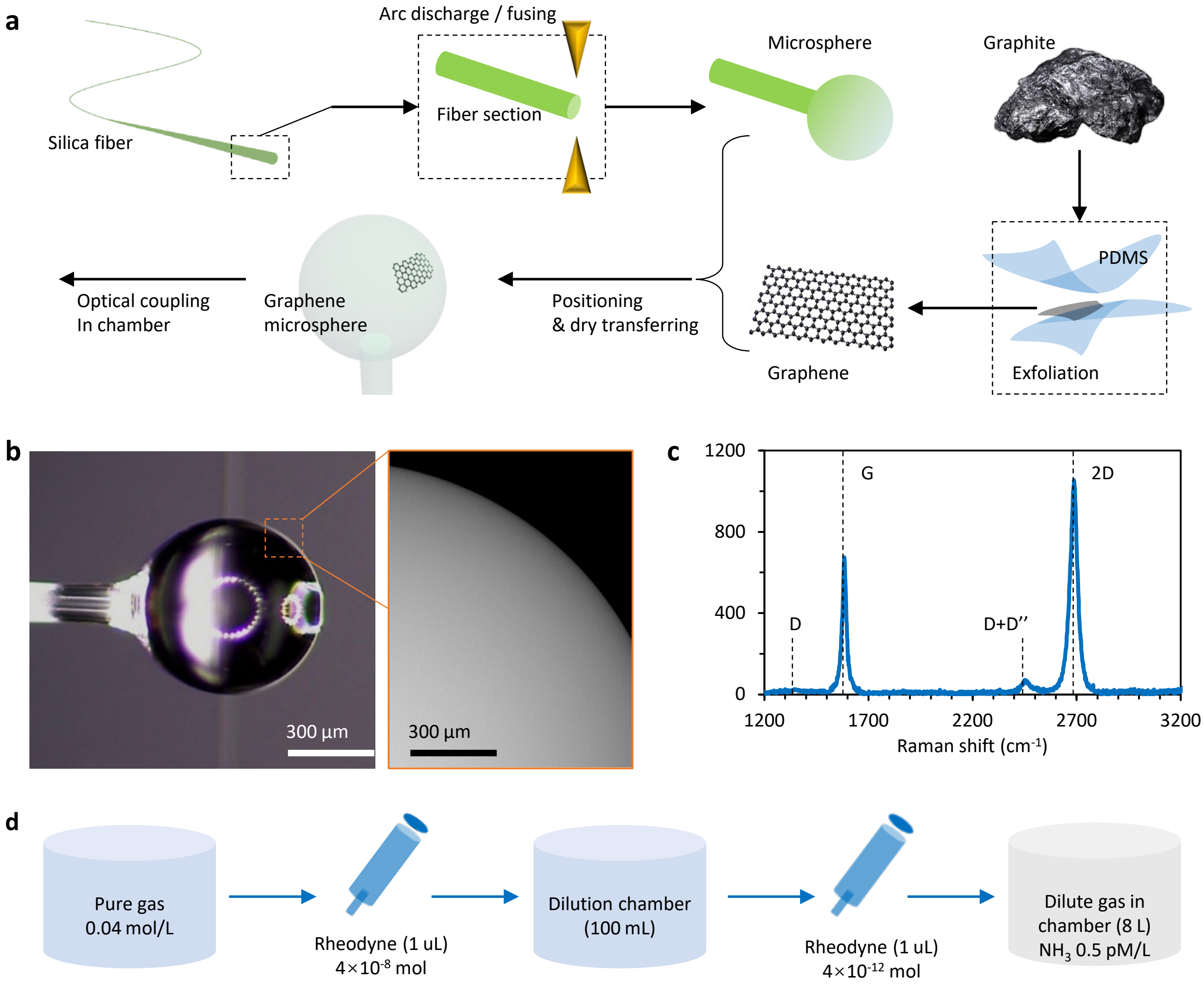

Fig. S3. Device fabrication and characterization. a, Fabrication process. i) we fabricate the silica microsphere via arc-discharging/fusing. ii) We prepare 2D graphene from graphite via mechanical exfoliation. iii) The graphene is assembled on the microsphere via dry transfer. **b,** Microscopic images of the microsphere sample, scale bars are marked. **c,** Raman spectrum of the mechanically exfoliated graphene deposited on the device. **d,** NH_3 gas preparation.

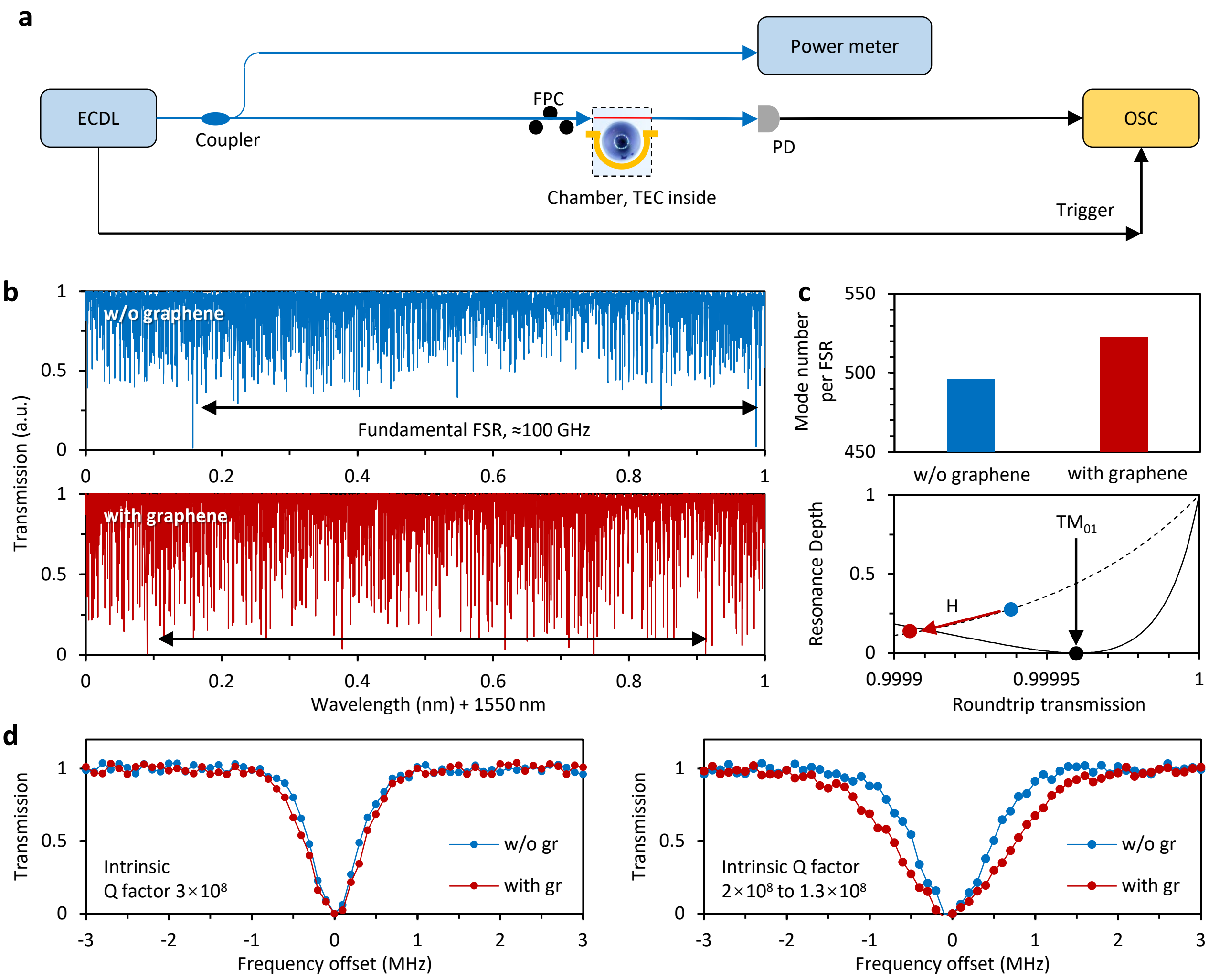

Fig. S4. Measured linear transmissions. a, Experimental setup for linear transmission measurement. ECDL: external cavity diode laser. PD: photodetector, OSC: oscilloscope. **b,** Linear transmission spectra in 1 nm span. Blue curve: before graphene deposition. Red curve: after graphene deposition. **c,** Top panel: Statistics of the resonance number in one FSR. Bottom panel: coupling curve of two modes. Solid curve: the graphene doesn't influence the fundamental mode. Dashed curve: graphene affects some higher order modes. **d,** Single resonance characterization. Left: the fundamental mode. Right: a higher order mode.

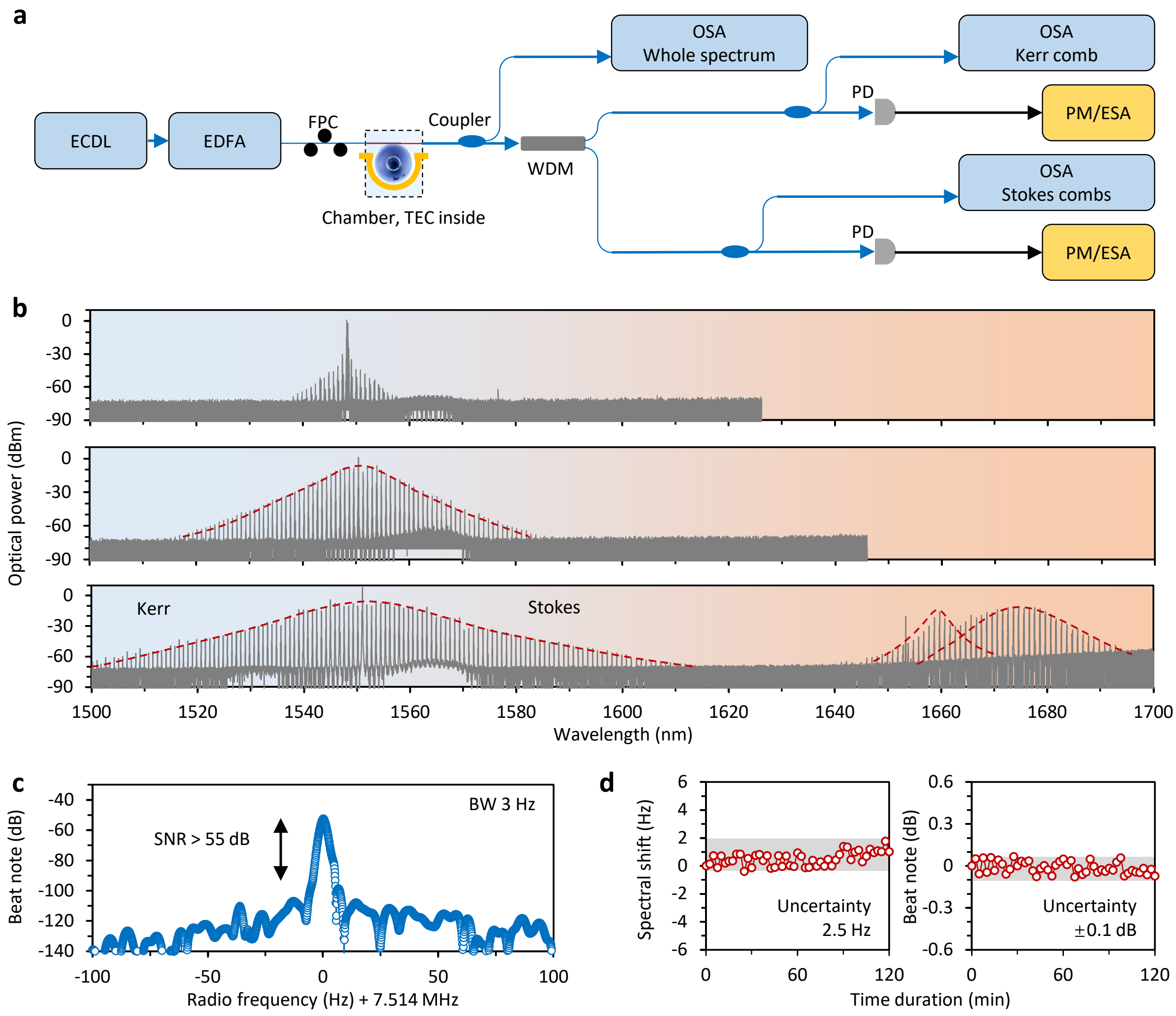

Fig. S5. Generation of the Kerr and Stokes solitons and inter mode comb beating. a, Experimental setup. **b,** Measured spectra during the comb generation, when increasing the pump red-detuning. **c,** One of the beat notes of Stokes solitons, with frequency 7.514 MHz. **d,** Its stability, measured in 2 hours. This shows > 55 dB SNR and < 10 Hz, also displays long-term (>2 hours) stability, with spectral uncertainty < 2.5 Hz, and intensity uncertainty < ±0.1 dB.

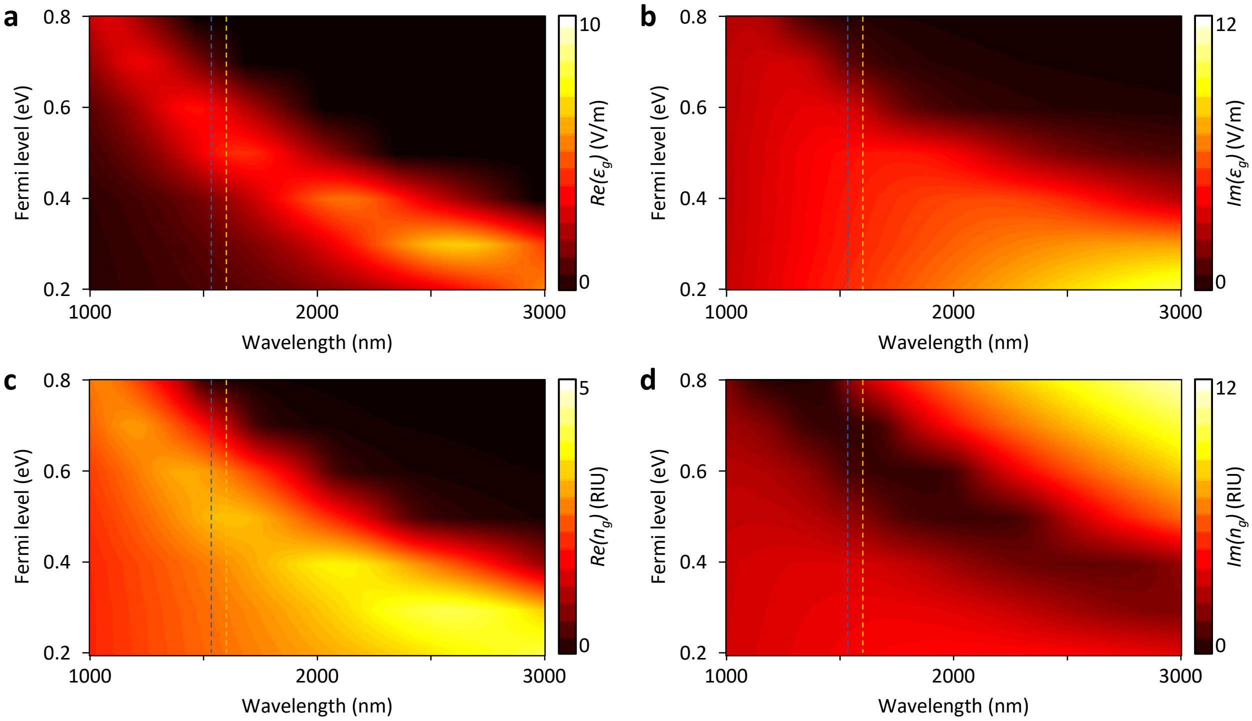

Figure S6 | Tunable optical refractive index of graphene. a and **b,** Real part and imaginary part of the graphene permittivity for E_F from 0.2 eV to 0.8 eV. **c** and **d,** Real part and imaginary part of the refractive index. Here the blue and yellow dashed lines mark the central wavelengths of the Kerr and Stokes comb.

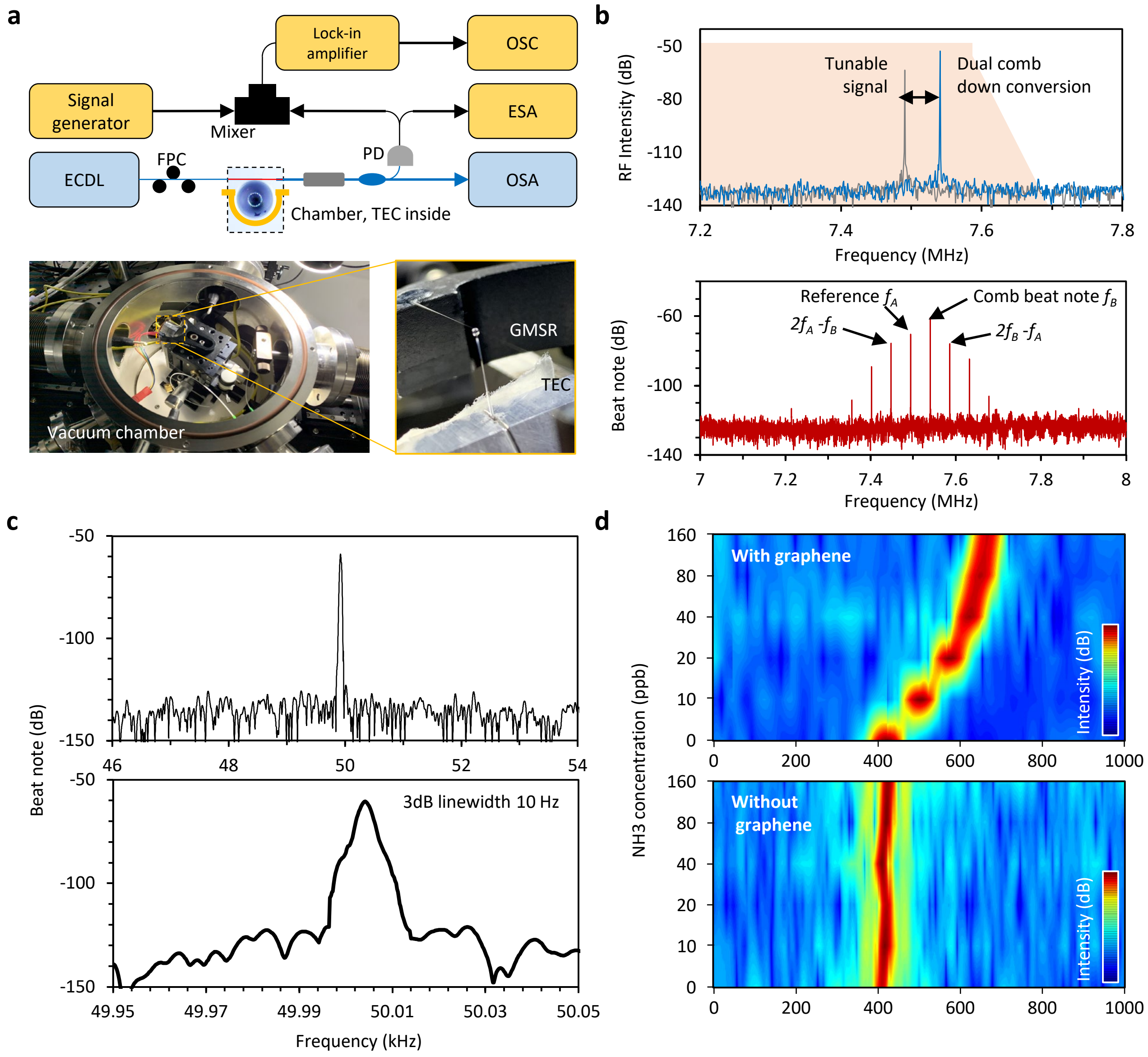

Fig. S7. Opto-electrical implementation for tracing individual gas molecule. a, Experimental setup. **b,** Top panel: Measured comb down conversion beat note (blue curve, @7.55 MHz) and a tunable electrical reference (grey curve, from a signal generator). Bottom panel: spectrum of the mixed two signals in the band 7 ~ 8 MHz, here we can see many harmonics. **c,** The mixing induced down conversion, which is used to detect the gas molecules. This signal is filtered and amplified in a lock-in amplifier. **d,** Comparison: graphene-based Stokes solitons show higher sensitivity to gas molecules, comparing with solitons generated in pristine silica microsphere.

Supplementary references

1. Agrawal, G. *Nonlinear Fiber Optics*. *Nonlinear Fiber Optics* (Elsevier, 2013). doi:10.1016/C2011-0-00045-5.

2. Liang, W. *et al.* Passively Mode-Locked Raman Laser. *Phys. Rev. Lett.* **105**, 143903 (2010).
3. Yang, Q.-F., Yi, X., Yang, K. Y. & Vahala, K. Stokes solitons in optical microcavities. *Nat. Phys.* **13**, 53–57 (2017).
4. Herr, T. *et al.* Temporal solitons in optical microresonators. *Nat. Photonics* **8**, 145–152 (2013).
5. Golovchenko, E. A., Mamyshev, P. V., Pilipetskii, A. N. & Dianov, E. M. Mutual influence of the parametric effects and stimulated Raman scattering in optical fibers. *IEEE J. Quantum Electron.* **26**, 1815–1820 (1990).
6. Kato, T. *et al.* Transverse mode interaction via stimulated Raman scattering comb in a silica microcavity. *Opt. Express* **25**, 857 (2017).
7. Novoselov, K. S. *et al.* A roadmap for graphene. *Nature* **490**, 192–200 (2012).
8. Ferrari, A. C. Raman spectroscopy of graphene and graphite: Disorder, electron–phonon coupling, doping and nonadiabatic effects. *Solid State Commun.* **143**, 47–57 (2007).
9. Yariv, A. Critical coupling and its control in optical waveguide-ring resonator systems. *IEEE Photonics Technol. Lett.* **14**, 483–485 (2002).
10. Mikhailov, S. A. & Ziegler, K. New electromagnetic mode in graphene. *Phys. Rev. Lett.* **99**, 016803 (2007).
11. Vakil, A. & Engheta, N. Transformation optics using graphene. *Science (80-.).* **332**, 1291–1294 (2011).
12. Lee, S. Colloidal superlattices for unnaturally high-index metamaterials at broadband optical frequencies. *Opt. Express* **23**, 28170 (2015).
